# Psychometric Characteristics of a Commuting-to-School Behaviour Questionnaire for Families

**DOI:** 10.3390/ijerph17228584

**Published:** 2020-11-19

**Authors:** María Jesús Aranda-Balboa, Marina Fernández, Emilio Villa-González, Berta Murillo-Pardo, José Manuel Segura-Díaz, Romina Gisele Saucedo-Araujo, Yaira Barranco-Ruiz, Manuel Herrador-Colmenero, Francisco Javier Huertas-Delgado, Palma Chillón

**Affiliations:** 1 PROFITH “PROmoting FITness and Health through Physical Activity” Research Group, Sport and Health University Research Institute (iMUDS), Department of Physical Education and Sports, Faculty of Sport Sciences, University of Granada, 18011 Granada, Spain; marinafc7@hotmail.com (M.F.); jmsegdia@ugr.es (J.M.S.-D.); rgs@ugr.es (R.G.S.-A.); mhc@ugr.es (M.H.-C.); pchillon@ugr.es (P.C.); 2 PROFITH “PROmoting FITness and Health through Physical Activity” Research Group, Sport and Health University Research Institute (iMUDS), Department of Physical Education and Sports, Faculty of Education and Sport Sciences, University of Granada, 52005 Melilla, Spain; evilla@ugr.es (E.V.-G.); ybarranco@ugr.es (Y.B.-R.); 3 Faculty of Education, University of Zaragoza, 50009 Zaragoza, Spain; murillop@unizar.es; 4 “La Inmaculada” Teacher Training Centre, University of Granada, 18013 Granada, Spain; fjhuertas@ugr.es

**Keywords:** active transportation, adolescents, children, parents, surveys

## Abstract

The purposes of this study were: (a) to describe the patterns of modes of commuting to school (children) and to work (parents) separated by gender and age, (b) to validate the questions on children’s mode of commuting to and from school according to their parents, and (c) to analyse the reliability of a family questionnaire focused on commuting to school behaviours. A total of 611 parents (mean age: 43.28 ± 6.25 years old) from Granada (Spain) completed “Family commuting-to-school behaviour” questionnaire in two sessions separated by 14 days, (2016 and 2018). The validation between family and children’s questions was assessed using the Kappa and Spearman correlation coefficients, and the test–retest reliability within the family questions was assessed using the Kappa and the weighted Kappa. The children’s modes of commuting to school (mean age: 11.44 ± 2.77 years old) were mainly passive (57.7% to school) while parents’ modes of commuting to work were mainly active (71.6%). The validity of the mode of commuting was significant with high Kappa and Spearman coefficients. The test–retest reliability presented a good agreement for the mode of commuting to school in children, distance and time to school, and the mode of commuting to work in parents, while the questions on acceptable distance to walk or cycle to school showed a moderate to good agreement. The “Family commuting-to-school behaviour” questionnaire could be a useful tool to assess the mode of commuting of children, distance and time to school for researchers and practitioners.

## 1. Introduction

Physical inactivity is the fourth most important global mortality risk factor [1]. The recommendations of physical activity (PA) for the youth are at least 60 min of moderate-to-vigorous PA per day [2,3]. Currently, there is a great concern about the children’s low PA level and the scientific literature has shown evidence about the decrease in these levels in children and adolescents in the last decades [3,4]. Physical activity during childhood and adolescence is associated with numerous health benefits such as improving musculoskeletal health [5], health-related quality of life [6], social relationships [7] and academic and cognitive performance [8].

Active commuting to school (ACS), i.e., walking or cycling to school as a daily behaviour, is a major active living domain as Sallis et al., reported [9], that contributes to increasing the PA levels [10,11] and cardiorespiratory fitness [12,13,14] and even reduces the incidence of metabolic syndrome [15] in children and adolescents. Therefore, ACS is presented as a low-cost solution with high viability to increase daily physical activity [16,17].

However, the prevalence of ACS has decreased from 2001 to 2013 in countries such as Australia (from 44% to 21%) [18], USA (from 41% to 13%) [19], and England (from 71% to 62%) [20]. Specifically, in Spain, the reduction of ACS in children was from 61% to 46% [21] from 2001–2002 to 2006–2007. Consequently, it is important to determine the factors that affect this behaviour in order to increase its prevalence.

According to the ecological framework developed by Mandic et al., there are three groups of factors (i.e., personal, social, and environmental) that determine ACS of the youth [22]. Firstly, in relation to the personal factors (e.g., socioeconomic level), families where the father or the mother is unemployed seem to have more active children [23], corroborating previous studies that affirm that ACS is associated with lower socioeconomic levels worldwide [24,25,26]. Secondly, as social factors (educational level), the type of school (public or private) and the mother’s educational level are predictors of ACS [27]. Thirdly, the environmental factor of distance from home to school is the main predictor of ACS with shorter distances (less than 1km) were positively associated with higher rates of ACS [24,28]. 

The relevance of ACS behaviour and the family involvement in it, makes necessary to research appropriate measurements for them, because different studies affirm that the parents’ decisions on the mode of commuting of their children are crucial [29,30]. Several studies have developed validated questionnaires in different contexts to assess ACS and its related determinants. A study carried out in the United States within the “Safe Routes to School Program” (SRTS) showed reliability from moderate to very high of a questionnaire for the parents about mode and frequency of ACS (24). Additionally, Adams et al. [31] obtained a moderate agreement in relation to the reliability and validity of PA measures in the commute; the questionnaire includes an assessment of travel behaviours in adults. In the same way, Bere et al. [32] reported test–retest reliability of a questionnaire about active commuting to school and work in 6th grade-school children and their parents in Norway. In Spain, there is only one study that shows parental perceptions of barriers to ACS, but it only focuses on the reliability of the parental barriers, where the study showed a good agreement for the questionnaire [33]. Therefore, it is necessary to develop a complete Spanish questionnaire version to assess children commuting to school behaviour from their parents’ responses.

Thus, the objectives of this study were: (1) to describe the patterns of modes of commuting to school (children) and to work (parents) separated by gender and age, (2) to validate the questions on the mode of commuting to/from the school of children according to their parents, and (3) to analyse the reliability of a family questionnaire focused on commuting to school behaviours. 

## 2. Method 

### 2.1. Participants and Procedure

A total of 611 parents (mother mean age: 42.63 ± 6.35 years old; father mean age: 45.19 ± 5.57 years old) and their 611 children (girls mean age: 11.52 ± 2.73 years old; boys mean age: 11.35 ± 2.83 years old) participated in this study completing a family and a student questionnaire, respectively. The data collection took place in two periods, between February and May 2016 and between March and April 2018, as part of the PACO (Pedalea y Anda al COle, Spanish acronym of Cycle and Walk to School) Study. The PACO Study examines ACS in Spanish children and adolescents and aims to develop strategies and interventions to promote adolescents’ ACS. The ethics committee of the University of Granada approved the study design, study protocols, and informed consent procedure (Reference: 162/CEIH/2016).

Firstly, the research team contacted seven different schools (five public and two private) of Granada selected by convenience. Initial meetings were conducted with the staff of the schools to communicate the information about the research project. Informed consent was delivered to parents through their children and they were signed by parents after the school acceptance of the research.

The “Family commuting-to-school behaviour” questionnaire was delivered to students, and they gave it to their parents or legal guardians to complete it as soon as possible (one week). The “Mode and frequency of commuting to and from school” questionnaire was completed by the students in the classroom with a researcher. Both questionnaires were completed twice in two sessions separated by 14 days. The research team emphasized that it was important that the retest family questionnaire was filled by the same person in the family that completed the test questionnaire. 

From the total of parents invited (*n* = 695), 611 completed the questionnaire in the first administration and only 230 questionnaires were completed in the second administration by the same parent who did it in the first administration. In addition, considering the difficulties to meet the parents, we decided to deliver the questionnaires through their children. Then, an additional difficulty was that parents did not understand why they had to complete it twice (33), so the sample is low in number. 

A total of 695 students from five public schools and two private schools of Granada were invited, where 611 completed the questionnaire at school.

For the first and second objectives, we included the parent sample that completed the questionnaire in the first assessment and had a corresponded child-questionnaire associated (611 parents and 611 children). For the third objective, we included a sample of 230 parents that had successfully completed the questionnaire in the two assessments by the same person (i.e., the father, mother, or legal tutor).

### 2.2. The “Family Commuting-to-School Behaviour” Questionnaire

To create the “Family commuting-to-school behaviour” questionnaire questions (Table 1), the Delphi Method [34] was used. It was developed in 5 phases:

(1) firstly, a deep review of the scientific literature was performed in order to find the family variables that may be associated with ACS;
(2) in the second phase, a search through specialized literature was conducted and the relevant papers focused on questionnaires about the mode of commuting were selected. Systematic research was conducted [35] to analyse different studies that used questionnaires in children, adolescents and both. In addition, several questionnaires [25,36,37] were analysed to elaborate the first version of the “Family commuting-to-school behaviour” questionnaire;
(3) in the next phase, independent active commuting experts were selected to evaluate the questionnaire. The experts focused mainly on the correct formulation of each item and the answers, in order to make it fully understandable. Finally, the questions were elaborated according to the experts’ ideas (questions on the mode of commuting of children and parents, questions on the distance and time to school, questions on the accompaniment of children in the journey to school and also questions on the acceptable distance to go to school on foot or by bike);
(4) in this phase, a pilot administration with parents was conducted. Suggestions made by parents were registered by researchers to improve the legibility of the items;
(5) the final version of the questionnaire was developed. It included nine questions divided into five categories (mode of commuting, accompaniment, distance, time, and permission for ACS) (Table 1).

The “Family commuting-to-school behaviour” questionnaire includes additionally a section with the personal data, consisting of variables such as gender, age, and socioeconomic status.

### 2.3. The “Mode and Frequency of Commuting to and from School” Questionnaire

In relation to the second objective of this study, we have used the “Mode and frequency of commuting to and from school” questionnaire [38]. This questionnaire showed a good convergent validity and reliability [38,39]. For the objective of this study, each child was paired with his/her parent that filled in the “Family commuting-to-school behaviour” questionnaire in the same administration. 

We used the questions related to ACS in “*the mode and frequency of commuting to and from school questionnaire*” to complete this objective: “*How do you usually go to school?*” and “*How do you usually go home from school?*”, for which the possible answers were *walking, cycling, car, motorbike, scholar bus, public bus, metro/train, or other*; only one option could be chosen. We only used both questions to report children’s mode of commuting to/from school.

### 2.4. Statistical Analyses

The descriptive data of the participants are presented as frequencies (and percentages) for the categorical variables and as mean (and standard deviation) for the continuous variables. Differences between mothers and fathers were calculated using the Student’s T-test for continuous variables and the chi-square test for categorical variables. Reliability was analysed using the kappa (to categorical variables) and the weighted kappa (to ordinal variables) coefficients. The results of the kappa and the weighted kappa were considered as: poor agreement (0–0.20), acceptable agreement (0.21–0.40), moderate agreement (0.41–0.60), substantial/good agreement (0.61–0.80) and almost perfect/very good agreement (0.81–1.00) [40]. As the parent is the person of authority, the children’s questions on mode of commuting were validated regarding the parents’ responses. The Kappa and the Spearman correlation coefficients were used to compare the parent and children’s responses. The Spearman correlation coefficients were interpreted as low (<0.30), moderate (0.30–0.50), and high (>0.50) [41]. All the analyses were performed with the statistical package SPSS for Windows version 23 (SPSS Inc., Chicago, IL, USA), establishing a level of statistical significance of *p* < 0.05. 

## 3. Results

The descriptive data of participants and the differences between the father and the mother are presented in Table 2 from “Family commuting-to-school behaviour” questionnaire. The children’s mode of commuting to/from school was mostly passive (57.7% and 56.4% respectively) and the accompaniment to/from school was mainly independent (76.3% and 73.0%, respectively). In addition, the distance and time from home to school were less than 2 km and less than 15 min (55.5%, and 57.1%, respectively). The parents’ mode of commuting was mainly active to work (71.6%, *p* < 0.001). The mean age of children was 11.44 ± 2.77 years old and, separated by gender, the mean age of girls was 11.52 ± 2.73 years old and in the case of boys, it was 11.35 ± 2.83 years old. 

Table 2 shows children’s mode of commuting separated by age (i.e., children vs. adolescents). Both groups of age presented mainly passive modes of commuting to school (children = 57.7% and adolescents = 53.1%). However, the adolescents showed a higher percentage of active vs passive mode of commuting from school, being significantly higher than children (*p* = 0.005). There were no differences between boys and girls in the mode of commuting.

The agreement of the children’s responses in relation to the parental response is presented in Table 3. The results showed a very good or almost perfect agreement (kappa coefficients range between 0.810–1.00) for the mode of commuting to/from school, even when they were separated by children and adolescents (children to school k = 0.864; adolescent to school k = 0.863), except for the mode of commuting from school in children that presented a good agreement (k = 0.806). In addition, there were high correlation coefficients for the mode of commuting to/from school for the total sample (to school, rho = 0.882; from school, rho = 0.860) and separated by age (children to and from school, rho = 0.862; rho = 0.839, respectively; adolescents to and from school, rho = 0.904; rho = 0.879, respectively). In relation to gender, the results showed a very good or almost perfect agreement in girls and boys for commuting to school (k = 0.881 and k = 0.846, respectively), a very good or almost perfect agreement for girls for commuting from school (k = 0.870) and a good agreement for boys for commuting from school (k = 0.799). Moreover, high Spearman coefficients were observed in both gender to/from school although they were higher in girls than boys: girls commuting to school, rho = 0.911; girls commuting from school, rho = 0.908; boys commuting to school, rho = 0.847; boys commuting from school, rho = 0.798.

The test–retest reliability analyses are shown in Table 4. Overall, the children’s mode of commuting, distance, and time to school, and the parents’ mode of commuting to work showed a good or almost perfect agreement (k = 0.951; k = 0.893; k = 0.850; k = 0.814, all *p* < 0.001), while the acceptable distance to walk or cycle on their own showed a good or moderate agreement (k = 0.771 and k = 0.692, respectively; all *p* < 0.001). In addition, the acceptable distance showed higher values of reliability for mothers than fathers.

## 4. Discussion

The main findings of the current study were that children’s mode of commuting to/from school was mainly passive, but more active in adolescents than in children. Meanwhile, parents’ modes of commuting to work were predominantly active, fathers being more active than mothers. The validation of the questions on the mode of commuting to/from school, reported by both parents and children, presented a very good or almost perfect agreement and high correlation coefficients. Regarding the reliability of the family questionnaire, results showed an almost perfect agreement in relation to the mode of commuting, including questions about distance and time, and a good agreement in questions about who accompanied the children to school; the questions about acceptable distance to go to school on foot or cycling showed a moderate agreement.

In our results, children mainly used passive modes of commuting, while adolescents are more active than children, similar to Herrador-Colmenero et al.’s study results [42] in the Spanish population. Surprisingly, the parents in this study presented very high rates of active commuting to work, being fathers more active than mothers. These results about commuting to work are higher than the study of Velde et al. [43] where 5.7% of the parents cycled and 18.2% walked to work at least 4 days per week. In relation to gender, no other studies presented these differences [44] between the percentages of parents’ mode of commuting to work. The parental results may be confirmed by future studies focused on parents, where the parents’ perceptions of their own mode of commuting could be reported. 

The validation of the questions about the mode of commuting to school was studied using parents and children’s responses to the same questions. The questions about the mode of commuting to/from school presented very good or perfect agreement and high values of correlation. Similar results were obtained in other studies carried out in other cities such as in Charlotte [25], in the region of Auckland, [36], or a study in the region of North Carolina [45]. In the study in Charlotte, the validity of the student–report travel mode was assessed by comparison with the parent–report one; in consonance with our study, a kappa coefficient with a good agreement for both modes was obtained as result [25]. In Auckland, the validity of the questions on children’s mode of commuting regarding parents was assessed with a similar sample to the one in our study; they obtained a kappa coefficient between 0.85–0.98, a very good or almost perfect kappa coefficient [36]. The study in the region of North Carolina showed a substantial agreement between parental and child reports for the mode of commuting (k = 0.80) [45]. After analysing the results obtained in this study and reviewing several articles, it is possible to affirm that we can obtain the mode of commuting through the “Family commuting-to-school behaviour” questionnaire and the “Mode and frequency of commuting to and from school” questionnaire, both being valid. 

In relation to the reliability of the family questionnaire, our results showed a very good or almost perfect agreement in the questions on the mode of commuting to/from school and work, as well as the distance and time. In the study of McDonald et al. [25], with a sample of 262 parents–students, they obtained results from moderate to very high agreement for the questions about travel mode and journey. On the other hand, in a study carried out in England in 2004, 103 adolescents were assessed on the means of commute to school; values of similar reliability coefficients were obtained (very good or almost perfect agreement) [46]. Finally, a study in Belgium with 33 adolescents showed kappa values changing from 0.44 and 1.00 in the variables referring to active commute to/from school [47], which showed lower values than our results.

While the studies that involve families are scarce, there are numerous studies on this topic that directly involve children through completing questionnaires or hands-up surveys. A piece of research carried out in Norway, where the purpose was to study the test–retest reliability of a questionnaire on the active commute to school and work according to the seasons of the year, showed high results for all modes of commuting with high Spearman correlation coefficients in children [32]. The fact that the questionnaire is completed either by the child or by the mother/father can influence the reliability of the questionnaire. According to the previously mentioned studies [25,46,47], we hypothesize that the reliability of the questionnaires on the mode of commuting to/from school completed by the children obtained a huge range of kappa coefficients. Meanwhile, in our study, the one completed by fathers and mothers obtained kappa coefficients with a very good or almost perfect agreement for the same variables. Therefore, those questionnaires completed by parents seem more reliable. 

In addition to the reliability, the results of this study showed a high agreement between all studied items except “acceptable distance to walk/cycle to school” where kappa values with moderate to good agreement were obtained. These differences could be presented because the questions about the acceptable distance on foot or bike are related to perceptions. McDonald et al. [25] found a low test–retest reliability when they asked parents if they would allow children to walk or cycle and, as well, about the barriers for allowing them to walk and cycle. Moreover, Forman et al. developed a similar instrument for parents. He asked parents the importance of different factors in their decisions to allow their children to walk/cycle and the results showed test–retest reliability from moderate to good agreement [37]. Even here, the results were strong enough to use the questions. 

In our study, some limitations can be pointed out. Firstly, the sample of the study is a convenience sample and the minimum sample size was not calculating. Secondly, the questionnaire was delivered by children to their home, and the sample of parents was low. Another limitation was the potential confusion that there might be between the behaviour variability (i.e., variability in scores associated with changes in behaviour from one week to the other) and the technical variability (i.e., variability in scores associated with the questionnaire design). A potential solution to increase the sample might be meeting the parents on a specific day to fill out the questionnaire using a similar procedure to the one used with the children in the classroom or deliver an incentive to motivate families as several studies reported. In order to highlight the strengths of the study, it must be considered that it is a pioneer questionnaire in Spain, as well as being the first reliability and validity study with Spanish families that contribute to propose a questionnaire about the patterns of children commuting to school to be used for the society.

## 5. Conclusions

In conclusion, the “Family commuting-to-school behaviour” questionnaire is a valid and reliable tool to be used in order to explore the Spanish parents’ opinions about their children’s mode of commuting to/from school behaviour. The validity of the questions on parents and children’s mode of commuting to/from school was high, thus using either the parents’ or the children’s questions will be recommended to assess children’s mode of commuting. In addition, the “Family commuting-to-school behaviour” questionnaire presented a high reliability to assess children’s mode of commuting to/from school, time and distance to the school, and good to moderate agreement for the accompanying variables and acceptable distance to walk and cycle to school in the Spanish population. 

The use of this valid and reliable questionnaire is recommended to researchers and public administration in order to understand the children and adolescents’ commuting to/from school behaviours and conduct effective intervention programs.

## Figures and Tables

**Table 1:** Questions of “Family commuting-to-school behaviour” questionnaire classified by categories.

CATEGORIES	QUESTIONS
**MODE OF COMMUTING**	- How does your child usually go to school? - How does your child usually come back from school? - How do you usually go to work?
**ACCOMPANIMENT**	- Does your child go accompanied by adults to school? - Does your child come back accompanied by adults from school?
**DISTANCE**	- How far from the school does your child live?
**TIME**	- How long does your child get to the school, since leaving home?
**PERMISSION TO ACS**	- What distance do you consider acceptable for your child to commute to school walking on their own, accompanied by children under 18 (friends, siblings, neighbours…) or accompanied by adults? - What distance do you consider acceptable for your child to commute to school by bicycle on their own, accompanied by children under 18 (friends, siblings, neighbours…) or accompanied by adults?

**Table 2:** Descriptive data of the “Family commuting-to-school behaviour” questionnaire and sociodemographic characteristics of the parent’s sample according to gender.

	All	Mothers	Fathers	*p*
**Parents age** (*n* = 417)	43.28 ± 6.25	42.63 ± 6.35	45.19 ± 5.57	**<0.001**
**Study level** (*n* = 207)				
Non-University	143 (69.1)	111 (71.6)	32(61.5)	0.174
University	64 (30.9)	44 (28.4)	20 (38.5)	
**Income** (*n* = 190)				
Low (<1999 €)	127 (66.8)	96 (69.1)	31 (60.8)	0.283
High (>1999 €)	63 (33.2)	43 (30.9)	20 (39.2)	
**Commuting to/from school behaviours of children**				
Mode of commuting to school (*n* = 582)				0.740
**Active**	246 (42.3)	186 (42.7)	60 (41.1)	
**Passive**	336 (57.7)	250 (57.3)	86 (58.9)	
Mode of commuting from school (*n* = 582)				0.760
**Active**	254 (43.6)	191 (44)	63 (42.6)	
**Passive**	328 (56.4)	243 (56)	85 (57.4)	
Accompaniment to school (*n* = 392)				0.909
Yes	76 (19.4)	56 (19.2)	20 (20)	
No	299 (76.3)	224 (76.7)	75 (75)	
Sometimes	17 (4.3)	12 (4.1)	5 (5)	
Accompaniment from school (*n* = 392)				0.425
Yes	86 (21.9)	61 (21)	25 (24.8)	
No	286 (73)	213 (73.2)	73 (72.3)	
Sometimes	20 (5.1)	17 (5.8)	3 (3)	
Distance to school (*n* = 598)				**0.030**
<2 km	332 (55.5)	**258 (58.1)**	**74 (48.1)**	
>2 km	266 (44.5)	**186 (41.9)**	**80 (51.9)**	
Time to school (*n* = 602)				**0.014**
<15 min	344 (57.1)	**269 (60)**	**75 (48.7)**	
>15 min	258 (42.9)	**179 (40)**	**79 (51.3)**	
**Parent’s mode of commuting to work** (*n* = 194)				**0.001**
Active	139 (71.6)	**95 (65.5)**	**44 (89.8)**	
Passive	55 (28.4)	**50 (34.5)**	**5 (10.2)**	
**Acceptable distance to walk to school** (*n* = 580)				
On their own				N/A
<2 km	186 (95.9)	136 (94.4)	50 (100.0)	
>2 km	8 (4.1)	8 (5.6)	0	
With children <18 years old				N/A
<2 km	175 (91.6)	129 (90.8)	46 (93.9)	
>2 km	16 (8.4)	13 (9.2)	3 (6.1)	
With an adult				0.948
<2 km	140 (71.8)	105 (71.9)	35 (71.4)	
>2 km	55 (28.2)	41 (28.1)	14 (28.6)	
**Acceptable distance to cycle to school** (*n* = 570)				
On their own				0.753
<2 km	161 (84.3)	119 (83.8)	42 (85.7)	
>2 km	30 (15.7)	23 (16.2)	7 (14.3)	
With children <18 years old				0.073
<2 km	145 (77.5)	113 (80.7)	32 (68.1)	
>2 km	42 (22.5)	27 (19.3)	15 (31.9)	
With an adult				0.066
<2 km	(106) 55.2	(85) 59	21 (43.8)	
>2 km	(86) 44.8	(59) 41	27 (56.3)	

N/A = Not applicable; Data in bold = Significant changes.

**Table 3:** Validation of the mode of commuting questions between parents and children.

	Complete Sample (*n* = 563)					
		Kappa	Rho			
Mode of commuting to school		0.865	0.882 **			
Mode of commuting from school		0.839	0.860 **			
	**Children (*n* = 311)**			**Adolescents (*n* = 252)**		
	***n***	**Kappa**	**Rho**	***n***	**Kappa**	**Rho**
Mode of commuting to school	309	0.864	0.862 **	250	0.863	0.904 **
Mode of commuting from school	304	0.806	0.839 **	248	0.867	0.879 **
	**Girls (*n* = 298)**			**Boys (*n* = 264)**		
	***n***	**Kappa**	**Rho**	***n***	**Kappa**	**Rho**
Mode of commuting to school	295	0.881	0.911 **	263	0.846	0.847 **
Mode of commuting from school	292	0.870	0.908 **	259	0.799	0.798 **

Notes: ** *p* value < 0.001.

**Table 4:** Test–retest reliability coefficients on commuting-to-school behaviours of children and fathers/mothers’ mode of commuting to work.

		All Participants			Mothers			Fathers	
	*n*	Kappa	*p*	*n*	Kappa	*p*	*n*	Kappa	*p*
Commuting to/from school behaviours of children									
To school	130	0.951	**<0.001**	104	0.939	**<0.001**	26	1.000	**<0.001**
From school	221	0.930	**<0.001**	175	0.929	**<0.001**	46	0.931	**<0.001**
Accompaniment to school	137	0.780	**<0.001**	110	0.724	**<0.001**	27	1.000	**<0.001**
Accompaniment from school	137	0.793	**<0.001**	109	0.753	**<0.001**	28	1.000	**<0.001**
Distance to school *	224	0.893	**<0.001**	177	0.889	**<0.001**	47	0.912	**<0.001**
Time to school *	227	0.850	**<0.001**	180	0.822	**<0.001**	47	0.777	**<0.001**
Parents’ mode of commuting									
To work	88	0.814	**<0.001**	69	0.812	**<0.001**	19	0.779	**<0.001**
Acceptable distance to walk to school *									
On their own	74	0.771	**<0.001**	58	0.856	**<0.001**	16	0.478	0.103
With children <18 years old	70	0.577	**<0.001**	54	0.610	**<0.001**	16	0.488	**0.003**
Adult	77	0.538	**<0.001**	60	0.532	**<0.001**	17	0.547	**0.005**
Acceptable distance to cycle to school *									
On their own	73	0.692	**<0.001**	57	0.733	**<0.001**	16	0.558	0.008
With children <18 years old	65	0.565	**<0.001**	50	0.526	**<0.001**	15	0.582	0.432
Adult	71	0.490	**<0.001**	55	0.455	**<0.001**	16	0.595	0.017

Notes: * Weighted Kappa; Data in bold = *p* value < 0.001.
